# Risky Decision-Making but Not Delay Discounting Improves during Inpatient Treatment of Polysubstance Dependent Alcoholics

**DOI:** 10.3389/fpsyt.2013.00091

**Published:** 2013-09-03

**Authors:** Bieke De Wilde, Antoine Bechara, Bernard Sabbe, Wouter Hulstijn, Geert Dom

**Affiliations:** ^1^Psychiatrisch Centrum Broeders Alexianen, Boechout, Belgium; ^2^Collaborative Antwerp Psychiatric Research Institute, Universiteit Antwerpen, Antwerp, Belgium; ^3^Universiteit Antwerpen, Antwerp, Belgium; ^4^Department of Psychology and Brain and Creativity Institute, University of Southern California, Los Angeles, CA, USA; ^5^Department of Neurology, University of Iowa, Iowa City, IA, USA; ^6^Clinical Research Division, Douglas Mental Health University Institute, Montreal, Canada; ^7^Radboud Universiteit Nijmegen, Nijmegen, Netherlands

**Keywords:** polysubstance dependence, impulsivity, risky decision-making, Iowa gambling task, treatment effects

## Abstract

**Background:** High levels of impulsivity, characteristics of addicted patients, are known to be important predictors of relapse. However, so far, little is known about the stability or variability of two main components of impulsivity (delay discounting and decision-making). The present study examined the changes in impulsivity during the first week of an abstinence based, behavioral orientated inpatient treatment program.

**Method:** Thirty-seven polysubstance dependent alcoholics completed the Delay Discounting Task (DDT), and the Iowa Gambling Task (IGT) using the original version with decks A′B′C′D′, and an alternative version with decks K′L′M′N′, for measuring decision-making, after 2 and 6 weeks of active treatment.

**Results:** It was found that performances on the IGT changed during treatment while performances on the DDT did not (test-retest period: 4 weeks).

**Conclusion:** The results provide preliminary evidence that improvements in decision-making might be related to treatment effects. All patients followed a highly structured cognitive-behavioral treatment program, which might have enhanced their executive functioning (coping skills training).

## Introduction

Addictions are chronically relapsing disorders characterized by compulsions to seek and take alcohol and/or drugs, loss of control in limiting intake, emergence of negative states, and a motivational withdrawal syndrome when access to the desired substance is prevented [e.g., (1, 2)]. A main element in this description, loss of control, involves a heightened impulsivity. The present study focuses on two manifestations of impulsive behavior: the inability to delay gratification, measured by the Delay Discounting Task [DDT; (3)], and a preference for risky decision-making, measured by the Iowa Gambling Task [IGT; (4)]. These elements are believed to be important in addiction [e.g., (5, 6)] and in relapse [e.g., (7–9)]. However, in spite of the assumed association between addiction and impulsivity, little is known about the stability or variability of delay discounting and decision-making in addicted patients over time.

In the past, both delay discounting and risky decision-making have been considered trait characteristics (i.e., individual specific, stable factors relatively independent of the environmental context). Consistent with this thought, only a few studies that used test-retest designs indicated that delay discounting was stable over time in healthy volunteers [e.g., (10–12)]. Studies in addicted patients were even fewer and less conclusive. While Takahashi et al. (13) found that DDT performances were stable over a 2-month period in abstinent alcoholics, Alfonso et al. (14) found IGT improvements in abstinent outpatients enrolled in a treatment program that included goal management training and mindfulness versus patients following Treatment as Usual (TAU) (test-retest period: 7 weeks). This last finding suggests that some impulsivity measures might be subject to change in addicted patients and specifically might be a good target for behavioral interventions.

The present pilot study was set up to explore changes of delay discounting and decision-making performances during the first weeks of an abstinence based, behavioral orientated inpatient treatment program. We focused on a sizeable group of polysubstance dependent alcoholics (PSA), a category of patients that are increasingly prevalent within inpatient treatment settings. In addition, this type of patients have been known to be highly impulsive and are characterized by severe performance deficits on behavioral decision-making tasks, i.e., DDT (3) and IGT (4). It has been shown that alterations in cognitive functions might occur soon after detoxification (15). Therefore, the tests and retests of DDT and IGT were completed early in abstinence, after 2 and 6 weeks of active treatment. Various clinical measures and self-report measures of impulsive personality were included to demonstrate the representativeness of our PSA group.

Based upon the limited number of earlier test-retest studies, we hypothesized that DDT performances would remain stable and that IGT decision-making would be ameliorated by the treatment effects.

## Materials and Methods

### Participants

**Figure 1:**
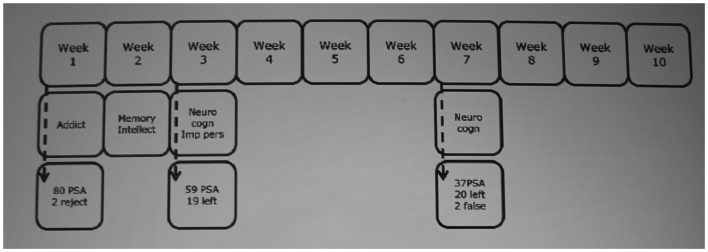
**Flow chart**. Addict: substance use measures (European Addiction Severity Index) – Memory (Rey Auditory Verbal Learning Test) – Intellect (Raven Progressive Matrices) – Neurocogn: neurocognitive measures (Iowa Gambling Task – Delay Discounting Task) – Imp pers: impulsive personality (Barratt Impulsiveness Scale – Sensitivity to Punishment and Sensitivity to Reward Questionnaires). PSA, polysubstance dependent alcoholics.

Eighty PSA consecutively enrolled in an addiction treatment program (Psychiatric Centre Broeders Alexianen, Boechout, Belgium) were asked to participate. Inclusion criteria were age (18–55) and lifetime dependence on three or more substances – Table 1 shows the patients substance use history, excluding caffeine and nicotine. Patients were excluded when they showed signs of lifetime psychotic disorders (sources: clinical psychiatric interview, Structured Clinical Interview for DSM disorders), had a history of prolonged coma or brain lesions, or low IQ (less than 80 – source: National Adult Reading Test). Two patients refused to participate and 19 left the addiction ward before baseline measures could be taken. Of the remaining 59 PSA, 20 left the addiction ward before administering the retests. Two patients were further excluded since their DDT data were unreliable. The flow chart can be found in Figure 1. Baseline measures were also obtained from 31 healthy controls (HC) without signs of lifetime psychiatric disorders.

**Table 1:** **Alcohol and drug use**.

	Age of onset	Years of substance use	Range
Alcohol (*n* = 31)	19.45 ± 5.74	11.48 ± 6.96	1–7.5 l (daily)
Cocaine (*n* = 21)	23.67 ± 7.41	5.95 ± 4.03	0,5–70 g (weekly)
Amphetamines (*n* = 26)	18.96 ± 5.25	7.19 ± 4.96	1–30 g (weekly)
Cannabis (*n* = 29)	17.00 ± 4.65	11.79 ± 7.43	5–25 g (weekly)
MDMA (*n* = 24)	20.25 ± 6.88	5.38 ± 4.14	5–30 tablets (weekly)

*Patients used on average 4.89 ± 2.01 substances. Substances are mentioned when at least half of the group abuses them*.

### Procedure

Polysubstance dependent alcoholics followed an inpatient, specialized, abstinence based addiction treatment program. An intensive, highly structured 8-week treatment program (group cognitive-behavioral therapy, motivational enhancement therapy, coping skills training, and stress management) followed a 2-week detoxification and diagnostic period. With the exception of withdrawal from pharmacotherapy (for a maximum of 10 days), the use of psychoactive medication was strictly limited. Abstinence was monitored using urine screening and breath analysis. Baseline measurement of impulsivity took place after detoxification (minimum controlled abstinence: 2 weeks) and retesting after 4 weeks of active treatment. HC completed baseline measurement of delay discounting and decision-making. The local ethical committee approved the study, and all participants provided informed consent.

Polysubstance dependent alcoholics completed the DDT and IGT as a part of a longitudinal outcome study examining the predicted value of different variables on relapse. The current test-retest data have not been previously reported.

### Settings and materials

Substance use measures
- The *European version of the Addiction Severity Index* [EuropASI; (16)] was used to assess PSA’s substance use 30 days preceding the treatment entrance.
- Self-report measures of impulsive personality
- The *Barratt Impulsiveness Scale* (BIS) (17), a self-report questionnaire (30 items), measured total, attentional, motor, and non-planning aspects of trait impulsivity.
- *The Sensitivity to Punishment and Sensitivity to Reward Questionnaires* (SPSRQ) (18) measured personality traits associated with the behavioral activation or appetitive system (sensitivity to reward) and the behavioral inhibition system (sensitivity to punishment).
- Cognitive measures of impulsivity
- *Delay Discounting Task* [computerized version, (3)]. Participants answered 100 questions, such as the following: “Would you rather have 10€ in 30 days or 2€ now?” A random adjusting amount procedure was used, so that the amount of immediate money was adjusted across trials until reaching an amount equivalent to a delayed reward, as determined by the participant’s choice. These indifference points were determined for all reward values (10€, 30€, 100€) and delays (2, 30, 180, 360, 720 days). Outcome measures were the mean logarithms of these delays for k at 10€, 30€, and 100€.
- *A*′*B*′*C*′*D*′*and K*′*L*′*M*′*N*′*versions of the Iowa Gambling Task* [IGT, (4)]. In both tasks, participants were instructed to select cards from four decks (A′B′C′D′or K′L′M′N′) to earn as much money as possible. Unknown to them, card selections came with different pay-offs: good decks led to net gains (modest wins – small losses) and bad decks to net losses (large wins – larger losses). Outcome measures were mathematical differences between the number of cards picked from the advantageous decks and the number of cards picked from the disadvantageous decks calculated for blocks of 20 cards. In addition, the net IGT score was calculated as the sum of the results over the five blocks.
  The principles of the alternative IGT version (with decks K′L′M′N′) are identical to the original task (with decks A′B′C′D′) except for one key change. In the original version the advantageous decks (C′ and D′) yield smaller immediate rewards than the disadvantageous decks (A′ and B′) 100% of the time. This percentage is reduced to 70% in the alternative version, so that 30% of the time, the advantageous decks yield rewards that resemble the average reward of the disadvantageous decks, and 30% of the time the disadvantageous decks yield rewards that resemble the average of the advantageous decks. This change circumvents the problem of improved performance due to repeated use in retest situations, when participants have experience with the original IGT and discover the rules of the task [e.g., (19)]. Upon learning the original IGT rules, a simple heuristic to succeed would be to avoid decks with higher initial gains. Therefore, in this IGT manipulation, 3 out of 10 cards from each deck would yield a gain that would contradict this simple heuristic. The net score of the alternative version is obtained by subtracting the total number of selections from the disadvantageous decks (L′ + N′) from the total number selections from the advantageous decks (K′ + M′). Evidence shows that normal subjects show similar scores to the original version when re-tested on the alternative version, i.e., no improved performance as a result of repeated use (19).

### Data analysis

Analyses of variance, following a General Linear Model procedure (GLM), were used to analyze DDT (within-subjects factors: test-retest and amount) and IGT performances (within-subjects factors: test-retest and block). Pearson correlation was used to explore the relation between clinical variables and IGT and DDT performances.

## Results

### Dropouts versus non-dropouts

Sociodemographic and substance use variables did not differ between groups [age: *t*(55) = 1.417; *p* = 0.162 – age of onset: *t*(55) = 1.409; *p* = 0.165 – years of substance use: *t*(55) = 1.414; *p* = 0.163 – EuropASI_Alcohol: *t*(55) = 0.026; *p* = 0.980 – EuropASI_Drugs: *t*(55) = −0.577; *p* = 0.566]. Dropouts however were less impulsive on the BIS than non-dropouts [BIS_Attention: *F*(1, 55) = 4.722; *p* = 0.034 – BIS_Motor: *F*(1, 55) = 2.151; *p* = 0.148 – BIS_Nonplanning: *F*(1, 55) = 4.564; *p* = 0.037 – BIS_Total: *F*(1, 55) = 6.463; *p* = 0.014]. Analyses of variance with Group (dropouts, non-dropouts) as between-subjects factor indicated that there were no differences on cognitive measures of impulsivity. GLM repeated measures analyses of variance with block (1–5) as within-subjects factor revealed that the IGT performances did not differ between groups *F*(1, 55) = 0.180; *p* = 0.673). There was a significant block effect [*F*(4, 52) = 3.477; *p* = 0.009], but no significant group × block interaction effect [*F*(4, 52) = 0.295; *p* = 0.881]. GLM repeated measures analyses of variance with amount (10€, 30€, and 100€) as within-subjects factor showed that DDT performances were similar between dropouts and non-dropouts [*F*(1, 55) = 0.345; *p* = 0.906]. There was a significant amount effect [*F*(2, 54) = 4.496; *p* = 0.013], but no significant group × amount interaction effect [*F*(2, 54) = 2. 178; *p* = 0.118]. Correlation analyses between time to drop out and self-report measures of impulsive personality on the one hand and cognitive measures of impulsivity on the other were weak and non-significant (range: −0.394–0.364).

### Descriptive measures

Sociodemographic variables did not differ between groups. PSA had more impulsive personality traits on both the BIS and the SPSRQ than did HC (Table 2).

**Table 2:** **Sociodemographic, self-report measures of impulsive personality, and cognitive measures of impulsivity (PSA, *n* = 37 – HC, *n* = 31)**.

	PSA (*n* = 37) week 3		PSA (*n* = 37) week 7		HC (*n* = 31)		*p*-Value PSA week 3 versus HC
		**Percent**				
Gender	29♂	8♀			27♂	4♀	0.321
	**Mean**	**SD**	**Mean**	**SD**	**Mean**	**SD**	
Age	31.61	6.87			28.06	7.79	0.052
Age of onset	15.78	4.00					
Years of substance use	14.33	5.82					
EuropASI_Alcohol	4.73	2.79					
EuropASI_Drug	5.30	1.70					
BIS							<0.001
BIS_Attention	19.03	4.39			14.83	3.77	
BIS_Motor	26.22	4.70			18.63	2.93	
BIS_NonPlanning	31.62	4.23			20.73	4.29	
BIS_Total	76.86	10.59			54.13	9.04	
SPSRQ							0.035
SPSRQ_SR	12.51	6.69			11.25	5.90	
SPSRQ_SP	13.03	4.30			10.36	3.74	
IGT							0.008
IGT_Block 1	−2.70	8.22	0.78	7.78	−2.19	8.40	
IGT_Block 2	1.68	9.09	4.54	8.06	4.39	9.68	
IGT_Block 3	2.32	10.33	4.27	9.20	6.13	9.48	
IGT_Block 4	0.97	9.82	3.59	10.27	5.65	9.38	
IGT_Block 5	0.00	9.47	2.22	10.28	8.58	10.34	
DDT							0.013
Log_k_10	−1.51	0.79	−1.51	0.74	−1.77	0.59	
Log_k_30	−1.57	0.72	−1.54	0.80	−2.00	0.54	
Log_k_100	−1.63	1.00	−1.68	0.97	−2.19	0.77	

*Substance use measures (PSA, n = 37). EuropASI, European Version of the Addiction Severity Index; BIS, Baratt Impulsiveness Scale; SPSRQ_SR, the Sensitivity to Reward Questionnaire of the Sensitivity to Punishment and Sensitivity to Reward Questionnaires; SPSRQ_SP, the Sensitivity to Punishment Questionnaire of the Sensitivity to Punishment and Sensitivity to Reward Questionnaires; IGT, Iowa Gambling Task; DDT, Delay Discounting Task*.

### Cognitive measures of impulsivity

Analyses of variance with Group (PSA, HC) as between-subjects factor indicated that PSA scored worse on both delay discounting and risky decision-making compared to HC (Table 2).

#### Iowa gambling task

**Figure 2:**
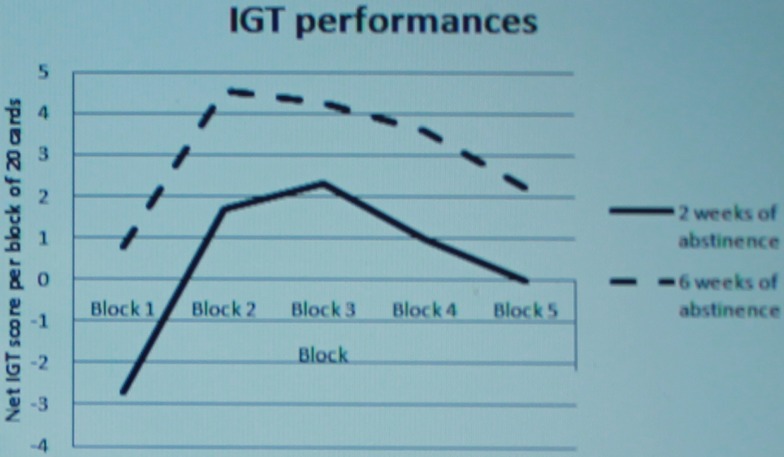
**Test and retest of the Iowa Gambling Task (PSA,*n* = 37)**.

General Linear Model procedure repeated measures analyses of variance with test-retest (2 and 6 weeks of abstinence) and block (1 to 5) as within-subjects factors revealed that the IGT performances changed from test to retest [*F*(1, 36) = 4.768; *p* = 0.036], and changed over blocks [*F*(4, 33) = 4.069; *p* = 0.004]. There was no significant test-retest × block interaction effect [*F*(4, 33) = 0.093; *p* = 0.984] (Figure 2). In addition, the IGT net score increased from 2.27 (baseline) to 15.41 (retest).

#### Delay discounting task

General Linear Model procedure repeated measures analyses of variance with test-retest and amount (10€, 30€, and 100€) as within-subjects factors showed that DDT performances were stable from test to retest [*F*(1, 36) = 0.321; *p* = 0.575]. Amount [*F*(2, 35) = 2.407; *p* = 0.105] or test-retest × amount interaction [*F*(2, 35) = 0.216; *p* = 0.807] effects were not significant.

### Correlations

Iowa gambling task net scores did not correlate [*r* = 0.25; *p* = 0.133] while test and retest DDT scores showed medium to high correlations [*r* = 0.32 (*p* = 0.056) up to 0.89 (*p* < 0.001)]. Correlations between IGT and DDT performances were not significant. Correlations of cognitive measures of impulsivity and sociodemographic with substance use variables including the duration of abstinence, were weak and non-significant. As expected, age correlated strongly with substance use duration [*r* = 0.63; *p* < 0.001] (Table 3).

**Table 3:** **Correlations between neurocognitive measures and other important measures**.

	Age	Age of onset	Duration	Abstinence	IGT	IGT 2	Logk_10_1	Logk_30_1	Logk_100_1	Logk_10_2	Logk_30_2	Logk_100_2
Age	1	0.27	0.63**	−0.25	0.06	−0.09	0.06	0.03	0.19	−0.16	−0.14	−0.17
Age of onset		1	−0.28	−0.05	0.15	0.11	0.22	0.20	0.23	−0.11	−0.08	−0.05
Duration			1	−0.22	−0.03	−0.07	−0.17	−0.39*	−0.26	−0.07	−0.18	−0.19
Abstinence				1	−0.07	0.28	0.15	0.06	0.09	0.18	0.15	0.10
IGT					1	0.25	−0.08	−0.16	−0.21	−0.09	−0.01	0.01
IGT 2						1	0.13	−0.09	0.11	−0.24	0.04	−0.08
Logk_10_1							1	0.75**	0.83**	0.43*	0.42*	0.41*
Logk_30_1								1	0.82**	0.52**	0.50**	0.54**
Logk_100_1									1	0.32	0.40*	0.41*
Logk_10_2										1	0.81**	0.87**
Logk_30_2											1	0.89**
Logk_100_2												1

Duration, years of substance dependence; Abstinence, days of abstinence; IGT, Iowa Gambling Task net score after 2 weeks of treatment; IGT 2, Iowa Gambling Tasks net scores after 6 weeks of treatment; logk_10/30/100_1, outcome measures Delay Discounting Task after 2 weeks of treatment; logk_10/30/100_2, outcome measures Delay Discounting Task after 6 weeks of treatment. The numbers in bold show the significant correlations (*p < 0.050; **p < 0.005).

## Discussion

The main finding of the present study was that decision-making, but not delay discounting, improved in PSA during behavioral inpatient treatment. PSA improved on the IGT while their DDT performances remained stable over time. This demonstrated improvement in IGT performances is conservative because in normal population the scores on the alternative version are similar to, and even slightly worse than the original IGT scores, due to the increased difficulty of the alternative task, which offsets any improvement in performance due to repeated use (19). Therefore, the improved IGT performances of PSA is significant and cannot be accounted for by practice alone.

The improvement of IGT performances in our inpatient PSA group occurred early in abstinence, after 4 weeks of therapy. Before considering possible consequences of this new finding for clinical practice, we will first discuss two alternative explanations of this result.

First, and as indicated earlier, better IGT performances at retest might be the result of a learning effect due to practice (or repeated use). This would imply that all subjects who complete a retest would show similar improvements. This explanation needs to be considered because in the present study, our design did not include a retest of the HC group. However, the IGT version used at retest (alternative version) was more difficult than the version used at baseline (original version), and in normal individuals, the scores on the alternative version are equal and even slightly worse than the scores on the original version, as shown in a recent normative study using these two versions of the IGT (19). This suggests that the alternative IGT version is insensitive to practice, and that the improved performances detected in PSA is due to treatment, and not practice alone.

A second alternative explanation is that the improvement might result from a process of natural recovery due to protracted abstinence. Indeed, it has been shown that (neuro)cognitive deteriorations following alcoholism or drug dependence withdrawal improved once substance use stopped [e.g., (20–22)]. However, research on the correlation between IGT performances and abstinence duration is inconclusive. Zhang et al. (23) for instance found that abstinence duration correlated with the IGT task (heroin dependent patients) while Verdejo-Garcia et al. (24) did not (polysubstance abusers). In addition, Fein et al. (25) showed severe IGT performances in long-term abstinent alcohol dependent patients. In the current study no significant correlation was found between abstinence duration and both IGT performances, indicating that natural recovery was unlikely.

A final and in our view also plausible hypothesis is in agreement with the position taken by Alfonso et al. (14) who suggested that treatment effects were responsible for the improvement in IGT performances because improvement was observed only in a group that participated in additional goal management training combined with mindfulness. The interactive goal management training was designed to improve executive coping, known to be important in decision-making, as measured by the IGT. Our patients might also have benefited from a highly structured cognitive-behavioral treatment program that focused on different coping strategies (e.g., planning, self-control, stress management, and relapse prevention).

In the current study, DDT performances did not change during inpatient treatment. This is consistent with previous research results (10, 11, 13). It suggests that delay discounting, apart from risk discounting, might be more of a personality trait. Alternatively, it might be that delay discounting is more important to the initiation of the addiction process (vulnerability factor) and less important to the latter stages of the addiction process and relapse.

Taken together, the results of our study provide further support to the hypothesis that decision-making might be variable. Reasons explaining the improvement remain to be explored. The effect might be due to practice (learning effect), natural recovery of the brain, treatment, and or interaction effects. Furthermore, it needs to be noted that the improvements in IGT performances in our study were substantial. The mean IGT net score changed from 2.27 to 15.41. A criterion net score of 10 was earlier used to define impairment (9, 26). This is of importance given the central role that impairments in decision-making may play in reducing the chances on recovery and abstinence. Indeed, an increasing number of studies reveal a close association between decision-making impairments and the risk on relapse (7, 9). Previously, several attempts have been made to enhance decision-making using pharmacological interventions. However, up to now results are inconsistent (27–29). The possibility of using psychosocial interventions may open a new window in developing new, more specific interventions. Our patients might also have benefited from a highly structured cognitive-behavioral treatment program that focused on different coping strategies (e.g., planning, self-control, stress management, and relapse prevention).

A main limitation of this study was the high percentage of dropouts, which is common for this type of research. *Post hoc* analyses showed that dropouts did not differ from non-dropouts in sociodemographic variables, addiction variables, delay discounting, and risky decision-making. Dropouts however showed less impulsivity than non-dropouts on measures of impulsive personality. These findings suggest that the remaining group was representative for a highly impulsive sample of PSA, indicating that this group might improve on IGT performances. A further limitation was the lack of control groups. Our findings cannot differentiate whether the improvements found are the consequence of therapy, abstinence, or their combination. To elucidate the possibility of natural recovery, i.e., sole effects of abstinence, future research should include abstinent PSA without formal treatment. However, within clinical settings this might prove to be difficult. Future research might reflect on including other settings such as prison-incarcerated individuals. Within the context of the causality question, the Alfonso et al. (14) data remain important showing that PSA without formal treatment showed less improvement on the IGT than PSA with formal treatment. Another limitation of this study is the relatively short period of treatment (week 7 of treatment) at which the patients were re-tested. However, the fact that within this short time frame we could find a significant improvement might suggest a powerful effect. It can be hypothesized that longer treatment periods would have allowed for even more substantial improvements. However the current study also possesses some important strengths, which include the selection of a sizeable, clinically relevant patient sample, and the close monitoring of abstinence (urine screening and breathalyzer) with respect to alcohol, illegal drug, or other psychoactive medication use during a highly structured inpatient treatment program.

## Conclusion

Our finding that decision-making within inpatient PSA improved to normal values after 4 weeks of structured treatment is new and should be further explored. However our study does not allow making formal conclusions concerning causality. Indeed, we cannot exclude other factors, e.g., abstinence are (partially) responsible for the improvements found. Future research needs to include a control group, i.e., abstinent patients without therapy, to allow examination of the effects of treatment, abstinence periods, and their interaction.

## Conflict of Interest Statement

The authors declare that the research was conducted in the absence of any commercial or financial relationships that could be construed as a potential conflict of interest.
